# Morphological characterisation of portal myofibroblasts and hepatic stellate cells in the normal dog liver

**DOI:** 10.1186/1476-5926-5-7

**Published:** 2006-11-16

**Authors:** Jooske IJzer, Tania Roskams, Ronald F Molenbeek, Ton Ultee, Louis C Penning, Jan Rothuizen, Ted SGAM van den Ingh

**Affiliations:** 1 Department of Pathobiology, Faculty of Veterinary Medicine, Utrecht University, The Netherlands; 2 Laboratory of Morphology and Molecular Pathology, University of Leuven (K.U. Leuven), Leuven, Belgium; 3 Department of Clinical Sciences of Companion Animals, Faculty of Veterinary Medicine, Utrecht University, The Netherlands; 4 TCCI Consultancy BV, PO Box 85032, 3508 AA Utrecht, The Netherlands

## Abstract

**Background:**

Hepatic fibrosis is a common outcome of hepatic injury in both man and dog. Activated fibroblasts which develop myofibroblastic characteristics play an essential role in hepatic fibrogenesis, and are comprised of three subpopulations: 1) portal or septal myofibroblasts, 2) interface myofibroblasts and 3) the perisinusoidally located hepatic stellate cells (HSC). The present study was performed to investigate the immunohistochemical characteristics of canine portal myofibroblasts (MF) and HSC in the normal unaffected liver as a basis for further studies on fibrogenesis in canine liver disease.

**Results:**

In the formalin-fixed and paraffin embedded normal canine liver vimentin showed staining of hepatic fibroblasts, probably including MF in portal areas and around hepatic veins; however, HSC were in general negative. Desmin proved to react with both portal MF and HSC. A unique feature of these HSC was the positive immunostaining for alpha-smooth muscle actin (α-SMA) and muscle-specific actin clone HHF35 (HHF35), also portal MF stained positive with these antibodies. Synaptophysin and glial fibrillary acidic protein (GFAP) were consistently negative in the normal canine liver. In a frozen chronic hepatitis case (with expected activated hepatic MF and HSC), HSC were negative to synaptophysin, GFAP and NCAM. Transmission electron microscopy (TEM) immunogold labelling for α-SMA and HHF35 recognized the positive cells as HSC situated in the space of Disse.

**Conclusion:**

In the normal formalin-fixed and paraffin embedded canine liver hepatic portal MF and HSC can be identified by α-SMA, HHF35 and to a lesser extent desmin immunostaining. These antibodies can thus be used in further studies on hepatic fibrosis. Synaptophysin, GFAP and NCAM do not seem suitable for marking of canine HSC. The positivity of HSC for α-SMA and HHF35 in the normal canine liver may eventually reflect a more active regulation of hepatic sinusoidal flow by these HSC compared to other species.

## Background

Hepatic fibrosis is a common outcome of hepatic injury in both man and dog. Depending on the primary site of injury the fibrosis may be restricted to the portal areas as in most biliary diseases or may be present in the hepatic parenchyma as seen in chronic hepatitis and cirrhosis. Chronic hepatitis is often diagnosed in pet-dogs. Treatment provides only limited results and the underlying mechanism of fibrosis is unclear. Activated fibroblasts which develop myofibroblasts (MF) characteristics play an essential role in hepatic fibrogenesis [1]. Three different MF-like cells have been described in rat and man based on location and immunohistochemical profile [2-4]. These comprise 1) portal or septal MF, present in the portal areas or in newly formed fibrous septa, 2) interface MF, present at the interface between parenchyma and stroma of the portal areas or newly formed fibrous septa, and 3) the perisinusoidally located hepatic stellate cells (HSC), also known as vitamin A-storing HSC, Ito-cells, hepatic lipocytes, lipid-laden cells, fat-storing cells or perisinusoidal lining cells. Debate exists regarding the origin of portal and interface MF and HSC. They may have a common origin in the primitive mesenchyme of the embryonal septum transversum. It remains to be elucidated which circumstances then lead to a different phenotype for the portal and interface MF and the HSC [5,6]. If stromal environment may promote transition and differentiation of HSC towards stromal MF, this might have therapeutic implications in patients.

Although portal and interface MF have been considered to have fibrogenic potential [7,8], most investigators regard the HSC as the principal fibrocompetent cell in the liver [5,9,10]. HSC are located in Disse's space, in between the hepatocytes and the sinusoidal endothelium, and play an important role in normal and diseased liver as they 1) produce the extracellular matrix, 2) act in a pericyte like manner around the sinusoids thus regulating sinusoidal blood flow, and 3) are the major site of vitamin-A storage in lipid vacuoles [9,10].

HSC have species-specific immunohistochemical expression profiles. All HSC express vimentin (rat), desmin (rat) and actin (man and rat), but alpha-smooth muscle actin (α-SMA) is classically considered as an indicator of activation (man and rat) [6,9,11]. However, in man α-SMA HSC reactivity proved to be strongly dependent on immunostaining conditions [12]. In addition to these myofibroblastic markers, human HSC also display some neuroendocrine features distinguishing them from the other hepatic MF-like cells in fibrotic liver [2]. They express synaptophysin [13], nerve growth factor (NGF), brain derived nerve growth factor (BDNF), neurotrophin-3 (NT-3), NT-receptors tyrosine kinase (Trk)-B and -C, and low-affinity nerve growth receptor p-75 (Trk-A), while other neuroendocrine markers as neural cell adhesion molecule (NCAM), glial fibrillary acidic protein (GFAP), NT-4, and alpha B-crystallin are expressed to a much higher extent in HSC than in the other hepatic MF subpopulations [2]. With parenchymal injury HSC transfer from a quiescent phenotype into an activated or transdifferentiated state characterised by increased proliferation, contractility and migration, as well as loss of vitamin-A containing lipid vacuoles and enhanced expression of α-SMA and desmin [9,11,14-16]. Furthermore, HSC produce growth factors as hepatocyte growth factor (HGF), transforming growth factor beta (TGF-β) as well as matrix metalloproteinases and abundant amounts of extracellular matrix components including collagen, proteoglycans and adhesive glycoprotein [5,9,16-18]. In dogs portal and interface MF and HSC have only been studied with α-SMA in activated HSC in a CCl_4 _intoxication model [19].

The purpose of this study was to investigate immunohistochemical characteristics of canine portal and interface MF and HSC in the normal unaffected liver, as a basis for further studies on fibrosis in canine liver disease.

## Results

### General observations

Routine haematoxylin and eosin (H&E) sections in all dogs revealed a normal liver. With large individual differences, presumptive vitamin A-storing HSC were regularly seen with a single large vacuole (vitamin A-storing lipid droplet) and a dislocated nucleus. HSC without a vitamin A-storing vacuole ("empty HSC") could not be identified on H&E sections. In immunostaining, negative controls were negative.

### Vimentin

**Figure 1:**
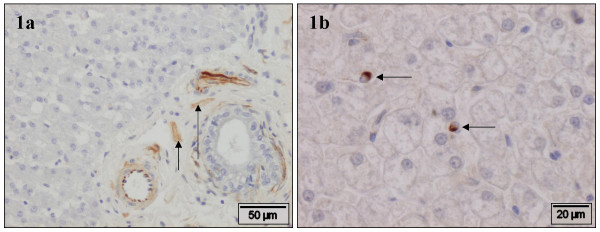
**Vimentin**. Normal canine liver, stained with vimentin antibody. **a) **In the portal area, smooth muscle cells of portal vasculature, most spindle-shaped stromal cells and neural cells (arrows) are positive. **b) **HSC were generally negative, although some individual positive cells are present (arrows).

There was strong variation between slides. In the portal area, vimentin showed positive staining cells in smooth muscle cells of portal vasculature, most spindle-shaped stromal cells and neural cells (Fig. 1a). Cells in Glisson's capsule as well as all stromal cells around the sublobular hepatic vein reacted positively to vimentin antibody. However, HSC were generally negative, although some individual positive cells were present (Fig. 1b).

### Desmin

**Figure 2:**
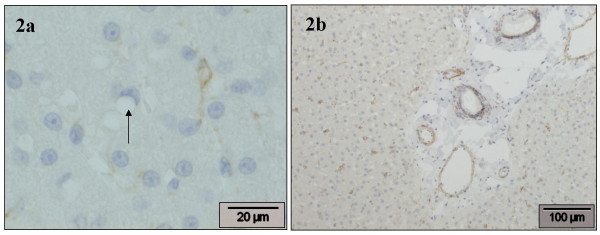
**Desmin**. Normal canine liver, stained with desmin antibody. **a) **A HSC (right) is positive in the perinuclear cytoplasm, weakly extending into a cytoplasmic process; also a negative vitamin A-storing HSC (arrow) is present. **b) **In the portal area, a moderate to strong staining is present in the smooth muscle cells of the arterial tunica muscularis and in the perivenular smooth muscle cells of the portal vein. HSC are weakly positive in the perinuclear cytoplasm.

There was also marked variation between slides. In general, HSC were weakly positive in the perinuclear cytoplasm, but vitamin A-storing HSC were predominantly negative (Fig. 2a). In the portal areas moderate to strong staining was present in the smooth muscle cells of the arterial tunica muscularis and in the perivenular smooth muscle cells of the portal vein (Fig. 2b). In addition, few positive spindle-shaped stromal cells were seen throughout the portal stroma and in the periductal location. In the sublobular hepatic veins smooth muscle cells were positive, in the surrounding stroma some MF appeared weakly positive. Incidentally, some positive cells were seen in Glisson's capsule.

### α-SMA

**Figure 3:**
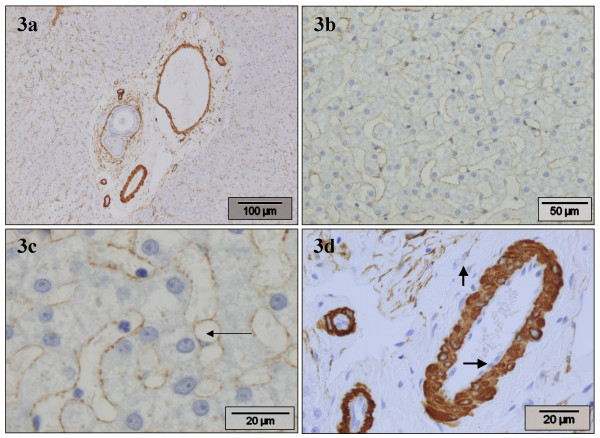
**α-SMA**. Normal canine liver, stained with α-SMA antibody. **a) **Portal areas show positivity around the bile ducts, in the arterial tunica media, and in the wall of the portal veins. There is slightly irregular moderate staining in the perisinusoidal spaces throughout the parenchyma. **b) **HSC stain positive, producing a thin irregular positive band lining the sinusoids. **c) **HSC stain positive. A positive cell containing one large vacuole (arrow-head is placed in vacuole) and a dislocated nucleus is seen. **d) **In the portal area there is strong positivity around the bile ducts and in the arterial tunica media, and moderate positivity in the wall of the portal veins, while endothelial cells remain negative (horizontal arrow). A portal MF with moderate positivity (vertical arrow) is present.

Consistently in all slides, this marker showed a slightly irregular (1–3 μm wide) moderate staining in the perisinusoidal spaces throughout the hepatic parenchyma (Figs. 3a,3b), and with higher magnification positive staining cells were observed containing small lipid vacuoles consistent with HSC (Fig. 3c). Vitamin A-storing HSC mostly showed positive cytoplasmic staining. Around the terminal and sublobular hepatic veins positive staining of pericytes and smooth muscle cells was observed, endothelial cells were consistently negative. Portal areas showed strong positivity around the bile ducts and in the arterial tunica media, and moderate positivity in the wall of the portal veins, while endothelial cells remained negative (Fig. 3d). Some portal MF, particularly in larger portal triads, showed weak to moderate α-SMA positivity (Fig. 3d). Glisson's capsule showed few positive cells.

### Muscle-specific actin clone HHF35 (HHF35)

**Figure 4:**
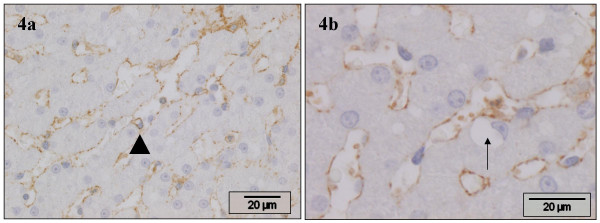
**HHF35 (muscle-specific actin, clone HHF35)**. Normal canine liver, stained with HHF35 antibody. **a) **HSC stain positive. Cells with few, small vacuoles stain positive (arrowhead). **b) **HSC stain positive. A vitamin A-storing HSC is negative (arrow).

Staining for this marker generally rendered similar results as α-SMA, consistently in all slides. In the portal areas, the terminal and sublobular hepatic veins, and in Glisson's capsule identical staining was observed. In the hepatic parenchyma moderate positive staining was seen in the HSC (Figs. 4a,4b). In comparison with α-SMA, HHF35 accentuated the perinuclear cytoplasm. As with α-SMA, regularly positive staining cells were seen with 1 to 3 small cytoplasmic lipid vacuoles (Fig. 4a). However, cells with a single large vitamin A-storing vacuole were mostly negative (Fig. 4b).

### GFAP, synaptophysin and NCAM

In formalin fixed normal canine liver tissue GFAP staining revealed few positive nerves located in larger portal areas (internal positive control) but no other positive staining was observed in any other location in these sections. Despite strong staining for synaptophysin in the adrenal medulla (external positive control), no staining was observed in any of the formalin fixed normal liver sections. In the frozen chronic hepatitis case (with expected activated hepatic MF and HSC), only nerves in larger portal areas reacted positively to GFAP and NCAM, while synaptophysin did not provoke any signal at all.

### Immunogold ultrastructural localization

**Figure 5:**
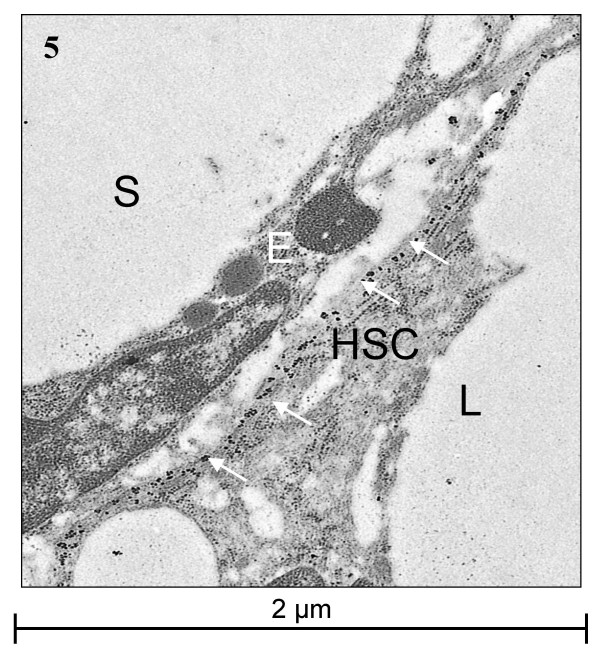
**TEM of α-SMA**. Immunogold labelling for α-SMA. The HSC is located in Disse's space between the endothelial cell and the hepatocyte. It has subendothelial cytoplasmic extensions and a prominent large lipid vacuole. The positive signal is present in the extensions (arrows). E = endothelial cell, HSC = hepatic stellate cell, L = lipid vacuole, S = sinusoid.

**Figure 6:**
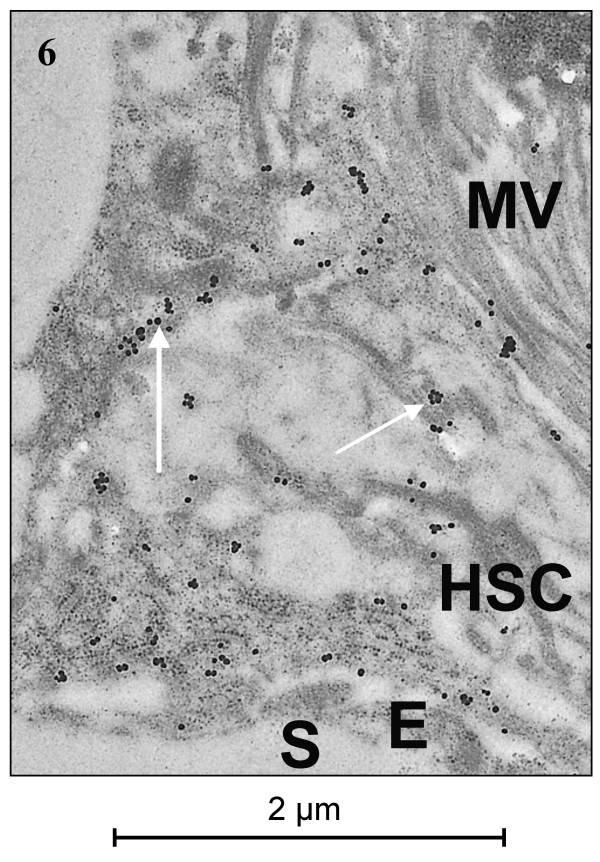
**TEM of HHF35 (muscle-specific actin, clone HHF35)**. Immunogold labelling for HHF35. The positive signal (arrows) is present in the subendothelial cellular extensions of the hepatic stellate cell situated in Disse's space. E = endothelial cell, H = hepatocyte, MV = hepatocytic microvilli, S = sinusoid.

Transmission electron microscopy (TEM) for α-SMA and HHF35 revealed a strong granular cytoplasmic staining restricted to subendothelial cells with long cytoplasmic extensions located in Disse's space (Figs. 5, 6). These cells often contained several smaller or one larger empty vacuole, interpreted as fat vacuoles (Fig. 5). Very slight, inevitable background staining was present in sinusoids and cells as small irregular spots, to be distinguished from the positive gold granules by a smaller and more irregular size. Both location and morphology of the positively staining cells identified them as HSC.

## Discussion

No antibody used in this study is species specific for the dog, but they still can be used due to interspecies cross-reactivity. All antibodies have been used previously in multiple other canine studies [20-26].

Variation in vimentin and desmin staining pattern was widely present. This might be due to the varying epitope sensibility, caused by the intrinsic patient material variability regarding time of postmortal sampling and fixation, the age of the paraffin blocks, and age, sex and breed variation of the animals. However, the used material reflects similar variability in intended patient populations to be studied for spontaneously occurring hepatic fibrosis, and thus provides useful insight in normal baseline variation.

In the formalin fixed paraffin embedded normal canine liver vimentin staining did not differentiate between fibroblasts and MF in the portal area and the perivenous stromal tissue. Moreover, HSC stained generally negative. Therefore, we conclude that vimentin antibody is not useful in paraffin sections as a marker for canine portal MF or HSC. Desmin stained MF in the portal area, around the sublobular hepatic vein and in Glisson's capsule. HSC stained inconsistently, with large variation between slides, so we conclude that desmin is not a sensitive marker for canine HSC. This is in contrast to man [6], but in accordance to rat [3].

In our laboratory, both α-SMA and HHF35 do identify myoepithelial cells in canine mammary gland. These monoclonal antibodies recognise different epitopes: a NH2 terminal decapeptide (α-SMA), and α and γ muscle actin (HHF35). The chance of formalin-induced epitope masking was regarded smaller by use of two different monoclonal antibodies for the same peptide. Therefore, both markers were investigated in related (regarding possible contractility) cells in the liver, being HSC and portal MF. In formalin fixed paraffin sections these cells can be easily identified in the normal canine liver by immunohistochemical staining for both α-SMA and HHF35. Both antibodies produced almost identical results and stained both solitary MF in the portal areas as well as HSC in the hepatic parenchyma. The presence of small lipid vacuoles in positively staining perisinusoidal cells as well as the TEM immunohistochemical results confirms the nature of the latter cells as HSC. The vitamin A-storing HSC usually stained positive for α-SMA but reacted only rarely to HHF35, suggesting differentiation in staining characteristics between less contractile vitamin A-storing HSC and more contractile HSC. The present finding of α-SMA reactivity which was diffusely present throughout the hepatic parenchyma in the normal canine liver is in contrast with findings in normal human and rat liver, where the majority of hepatic lobules are devoid of α-SMA positive HSC, or only show weak positivity [2,6,9,12]. In our opinion, this indicates a species-specific expression pattern for the dog. Rat and human HSC upregulate α-SMA expression upon activation [2,6,28] and this positive staining of activated HSC is considered to represent increased contractility of the cells [29,30].

Debate still exists regarding the contribution of non-activated quiescent HSC to sinusoidal blood flow and blood pressure in man and rat [9,11]. Our findings of α-SMA staining in HSC of normal dogs may suggest a more active role in controlling microvascular blood flow compared to the rat. Interestingly, the dog is unique in that it has a spirally shaped smooth muscle surrounding the sublobular hepatic veins consistent with a more active regulation of the hepatic blood flow in the dog compared to other species [31].

Despite positive staining of HSC for α-SMA in normal dogs reflecting contractility we feel it appropriate to regard these cells as "quiescent" HSC. This is in line with other species as HSC are most likely not activated in the sense of enhanced matrix- or TGF-β production. In the dog discrimination between quiescent and activated HSC does not seem possible with antibodies directed against α-SMA and HHF35. However, morphological changes or functional changes such as increased cell size, loss of lipid vacuoles and enhanced production of TGF-β and other substances may be helpful.

The absence of reactivity of portal MF and HSC in the normal canine liver to synaptophysin and GFAP indicates that in contrast to man and rat [2,3,6], canine portal MF and HSC do not seem to express the used markers in the normal liver. Moreover, in frozen sections from a dog with chronic active hepatitis which likely contained activated MF and HSC, these cells did also not react to antibodies for neural crest markers NCAM, GFAP and synaptophysin. As in frozen samples antigens retrieval is not necessary, we conclude that not only in the quiescent state but also in the activated state, canine portal MF and HSC do not bind the used antibodies for NCAM, GFAP and synaptophysin. Although probably limited by species-specificity of the antibodies, this study could be expanded by the use of more frozen tissue samples, or by the use of other potential markers, reacting positively on hepatic MF in other species, like NGF, BDNF, NT-3, NCAM [2], Foxf1 [32] or fibulin-2 [33].

## Conclusion

In formalin fixed paraffin sections, canine portal MF and HSC can be identified by α-SMA, HHF35 and to a lesser extent desmin immunostaining. In contrast to man, these cells are consistently negative for synaptophysin, GFAP and NCAM, both in formalin-fixed paraffin embedded tissue, as well as in frozen sections. Alpha-SMA and HHF35 positivity of HSC in the normal canine liver may reflect a more active regulation of hepatic sinusoidal flow by these cells compared to other species. Alpha-SMA and HHF35 can be used for further studies on hepatic fibrosis in the dog.

## Materials and methods

### Dogs

Normal liver tissue was obtained from ten dogs for immunohistochemistry: either patients with liver unrelated pathology (n = 8) or normal control animals euthanized for liver-unrelated research projects (n = 2). Laboratory exams regarding liver function were not performed. One frozen sample of a dog with chronic hepatitis was additionally used. Patients were submitted for their individual diagnostic purposes to the Department of Clinical Sciences of Companion Animals, or to the Department of Pathology, Faculty of Veterinary Medicine, Utrecht University. No tissue was taken purposely for the reported study. Projects were approved by the responsible ethical committees for the use of experimental animals and for use of client-owned animals according to Dutch legislation. After euthanasia as part of the research projects, we were allowed to take liver tissue of the two control animals. Included were six females and four males. Mean age was 13 months (± 15 months).

### Immunohistochemistry

Liver specimens were taken within 1 hour post mortem (n = 9), or in a surgical biopsy procedure (n = 1). The normal liver samples were fixed in 10% neutral buffered formalin and routinely embedded in paraffin, while the chronic hepatitis sample was snap-frozen in liquid nitrogen cooled isopentane and stored at -70°C. Sections (3 μm) were stained with haematoxylin and eosin for routine histology. Immunohistochemistry was performed for α-SMA, HHF35, desmin, vimentin, GFAP and synaptophysin on all normal liver sections, the frozen sections (chronic hepatitis) were subjected to GFAP, NCAM and synaptophysin immunohistochemical staining. Antibody characteristics, manufacturer, source and dilution are given in Table 1. For this purpose, sections (3 μm) were mounted on poly-L lysine coated slides and stored for a maximum of 48 hours at room temperature until use. After that slides were deparaffinized. Endogenous peroxidase activity was blocked by 1% H_2_O_2 _in methanol for 30 min at RT. As the protocols for demonstration of desmin and synaptophysin required an antigen retrieval step [13,21] sections were treated by heating in 10 mM citrate pH 6.0 in a microwave oven for 10 min, cooled down for 10 min at room temperature (RT). After washing with PBS buffer containing 0.1% Tween-20, background staining was blocked by incubating the sections with normal horse serum (1:10 diluted) for 15 min at RT for α-SMA, HHF35, synaptophysin and vimentin. Desmin and GFAP sections were blocked with normal goat serum (1:10 diluted) for 15 min at RT. Sections were incubated 60 min at RT with the primary antibody to α-SMA, desmin, GFAP or HHF35, and 30 min at RT for vimentin, while sections for synaptophysin were incubated overnight at 4°C. After washing in PBS-Tween, slides to be marked with mouse antibodies were incubated in horse-anti-mouse biotin (Vector Laboratories, Burlingame, CA, USA) (1:125 diluted) for 30 min at RT. GFAP and desmin sections were incubated in goat anti rabbit biotin (1:250 diluted) for 30 min at RT. After washing in PBS-Tween, sections were incubated in avidin-biotin peroxidase complex (Vector Laboratories). The colour was developed in 3-3'-diaminobenzidine, sections were counterstained with 10% Mayer's haematoxylin. Negative controls consisted of omission of the primary antibody, and replacement by non-immune serum. Formalin fixed paraffin embedded canine adrenal medulla served as positive control tissue for synaptophysin. The other antibodies had internal controls: for GFAP this were nerves in the larger portal tracts, while for α-SMA, desmin, HHF35 and vimentin arterial smooth muscle cells served as positive control tissue.

**Table 1:** Details concerning the used antibodies.

**Antibody**	**Manufacturer**	**Catalogue no**.	**Type**	**Clone**	**Dilution**	**Fixative**
α-SMA	BioGenex	MU 128-UC	mouse monoclonal	clone1A4	1:1200	formalin
Desmin	Eurodiagnostica	2203PDE	rabbit polyclonal	-	1:80	formalin
GFAP	ICN Biomedicals	10555	rabbit polyclonal	-	1:80	formalin
GFAP	Biogenex		mouse monoclonal	-	1:40	frozen
Muscle actin	Dako	M0635	mouse monoclonal	HHF35	1:300	formalin
NCAM	Chemicon		rabbit polyclonal		1:50	frozen
Vimentin	Dako	M0776	mouse monoclonal	SY38	1:100	formalin
Synaptophysin	Dako		rabbit polyclonal	-	1:50	frozen
Vimentin	Biogenex	MU074-UC	mouse monoclonal	V9	1:150	formalin

### Immunogold ultrastructural localization

For TEM, additional liver samples were taken from two female dogs, three and seven years old. Both were normal control animals euthanized for liver-unrelated research projects. Projects were approved by the responsible ethical committees for the use of experimental animals as required under Dutch legislation. After euthanasia as part of the projects, we were allowed to take liver tissue. Liver samples were taken immediately postmortem, fixed in 4% paraformaldehyde for 2 days, subsequently washed and transferred in methanol to an auto freezing device from Reichert. Freeze substitution was performed 36 h at -85°C in methanol, temperature was gradually raised in 5°C-steps to -45°C, followed by serial substitution from methanol to Lowicryl HM20 from methanol:HM20 = 2:1 (2 × 1 h) to methanol:HM20 = 1:2 (2 × 1 h) and pure HM20 2 h at -45°C. Polymerisation was performed for 36 h at -45°C, then temperature was raised in 5°C-steps for 13 h up to 20°C. Temperature stayed 20°C for 150 h all under UV-light. After ultrathin sectioning grids were labelled according to the procedure for single labelling. Free aldehyde groups were blocked in 50 mM glycine in PBS for 15 min, followed by 30 min aurion blocking solution for goat gold conjugates and washed in BSA-c buffer (PBS + 0.1% BSA-c, pH 7.4), 3 × 5 min. Overnight incubation of the primary antibodies α-SMA and HHF35 diluted in BSA-c buffer (1:1200 and 1:300 respectively) was followed by BSA-c buffer wash (6 × 5 min) and incubation of goat-anti-mouse IgG ultra small gold diluted 1:50 in BSA-c buffer (2 h). BSA-c buffer wash (6 × 5 min) and PBS wash (3 × 5 min) was done previous to postfixation in 2% glutaraldehyde in PBS (5 min), followed by wash in PBS (5 min) and distilled water (5 × 2 min). Signal enhancement was done using Aurion R-Gent SE-EM (30 min), and subsequent washing in distilled water (5 × 2 min). Grids were then stained with uranyl acetate and lead citrate. For sample evaluation a Philips CM 10 TEM was used.

## Competing interests

The author(s) declare that they have no competing interests.

## Authors' contributions

JY histochemically examined all slides and TEM findings, and wrote the manuscript. TR participated in study design and coordination and helped to draft the manuscript. RM stained all slides immunohistochemically. TU performed TEM studies. LP and JR conceived the study, participated in its design and helped to draft the manuscript. TI examined the slides and TEM findings, participated in its design and coordination and helped to draft the manuscript. All authors read and approved the final manuscript.
